# WWOX protein expression varies among ovarian carcinoma histotypes and correlates with less favorable outcome

**DOI:** 10.1186/1471-2407-5-64

**Published:** 2005-06-27

**Authors:** María I Nunez, Daniel G Rosen, John H Ludes-Meyers, Martín C Abba, Hyunsuk Kil, Robert Page, Andres JP Klein-Szanto, Andrew K Godwin, Jinsong Liu, Gordon B Mills, C Marcelo Aldaz

**Affiliations:** 1 Department of Carcinogenesis, The University of Texas M.D. Anderson Cancer Center, Science Park-Research Division, Smithville TX, USA; 2 Department of Pathology, The University of Texas M.D. Anderson Cancer Center at Houston, TX, USA; 3 Molecular Therapeutics, The University of Texas M.D. Anderson Cancer Center at Houston, TX, USA; 4 Department of Pathology, Fox Chase Cancer Center, Philadelphia, PA, USA; 5 Ovarian Cancer Program, Fox Chase Cancer Center, Philadelphia, PA, USA

## Abstract

**Background:**

The putative tumor suppressor *WWOX *gene spans the common chromosomal fragile site 16D (FRA16D) at chromosome area 16q23.3-24.1. This region is a frequent target for loss of heterozygosity and chromosomal rearrangement in ovarian, breast, hepatocellular, prostate carcinomas and other neoplasias. The goal of these studies was to evaluate WWOX protein expression levels in ovarian carcinomas to determine if they correlated with clinico-pathological parameters, thus providing additional support for WWOX functioning as a tumor suppressor.

**Methods:**

We performed WWOX protein expression analyses by means of immunobloting and immunohistochemistry on normal ovaries and specific human ovarian carcinoma Tissue Microarrays (n = 444). Univariate analysis of clinical-pathological parameters based on WWOX staining was determined by χ^2 ^test with Yates' correction. The basic significance level was fixed at *p *< 0.05.

**Results:**

Immunoblotting analysis from normal ovarian samples demonstrated consistently strong WWOX expression while 37% ovarian carcinomas showed reduced or undetectable WWOX protein expression levels. The immunohistochemistry of normal human ovarian tissue sections confirmed strong WWOX expression in ovarian surface epithelial cells and in epithelial inclusion cysts within the cortex. Out of 444 ovarian carcinoma samples analyzed 30% of tumors showed lack of or barely detectable WWOX expression. The remaining ovarian carcinomas (70%) stained moderately to strongly positive for this protein. The two histotypes showing significant loss of WWOX expression were of the Mucinous (70%) and Clear Cell (42%) types. Reduced WWOX expression demonstrated a significant association with clinical Stage IV (FIGO) (p = 0.007), negative Progesterone Receptor (PR) status (p = 0.008) and shorter overall survival (p = 0.03).

**Conclusion:**

These data indicate that WWOX protein expression is highly variable among ovarian carcinoma histotypes. It was also observed that subsets of ovarian tumors demonstrated loss of WWOX expression and is potentially associated with patient outcome.

## Background

The *WWOX *gene, originally cloned by our laboratory, spans a genomic region greater than 1 Mb in size and is the second most common chromosomal fragile site, FRA16D (16q23) [1,2]. Abnormalities affecting *WWOX *at the genomic and expression level have been reported in numerous neoplasias and cancer derived cell lines including, breast, ovarian, esophageal, lung, stomach, liver, pancreas and hematological malignancies [3-12]. We observed that ectopic WWOX expression inhibited anchorage independent growth and *in vivo *tumorigenicity of highly aggressive breast carcinoma lines, suggesting a putative tumor suppressor role for this novel protein [13,2].

*WWOX *encodes a 46 KD, 414-amino acid protein that contains two WW domains at the NH_2 _terminus and a short chain oxidoreductase (SDR) central domain [1]. The first WW domain- is involved in protein-protein interactions by binding the specific proline rich motif PPXY and several potential candidate partner proteins have been postulated [14,15]. Within the SDR domain, the presence of WWOX amino acid residues, serine 281, and 293-**Y**NRS**K**-297 make up a catalytic signature motif conserved in short-chain steroid dehydrogenases [16]. We originally reported high *WWOX *mRNA expression levels in ovary, prostate, testis and breast [1]. In this study we analyzed WWOX protein expression pattern in normal ovary and ovarian carcinomas. We correlated WWOX protein expression with ovarian carcinoma histotypes and clinico-pathological parameters. In addition, since we recently observed a strong association between loss of WWOX expression and estrogen and progesterone receptor (ER and PR) status in breast cancer [12], we also investigated any potential association between expression of sex steroid hormone receptors and WWOX in ovarian cancer.

## Methods

### Western blot analysis

Total protein extracts were prepared from snap frozen tissue of 38 human ovarian carcinomas and 5 normal human ovarian tissues. As negative control for WWOX protein expression we used protein extracts from the ovarian cell line PEO1 that does not express WWOX due to a homozygous deletion affecting exons 4–8 of this gene [4], a kind gift of Dr. Hani Gabra at Imperial College London, UK. As positive control we used the same cell line stably transfected with a WWOX expressing vector (PE01-WWOX) [10,12]. Total cell protein lysates were made using RIPA buffer (50 mM Tris pH7.5, 150 mM NaCl, 0.5% sodium deoxycholate, 1% Triton X-100, 0.1% SDS) containing protease inhibitor cocktail (Roche, Mannheim, Germany). For western blotting 50 ug of total protein was separated by 12.5% SDS-PAGE and transferred to PVDF membranes (Millipore, Billerica, MA). Immunodetection was performed using Protein Detector™ (KPL, Gaithersburg, MD) western blotting reagents as described by the manufacturer. WWOX protein was detected using affinity-purified anti-WWOX rabbit polyclonal primary antibodies developed in our laboratory (final concentration 280 ng/ml) [12] and HRP conjugated anti-rabbit secondary antibody (KPL, city, state, 1:2000 dilution) followed by chemiluminescence autoradiography. Actin was used as the protein loading control and it was detected using monoclonal anti-actin antibody (ICN biomedicals, Burlingame, CA, 1:1000 dilution) and HRP conjugated anti-mouse secondary antibody (KPL, 1:5000). Quantitation of X-ray films exposed to western blot chemiluminescence-emitting membranes was performed using a Kodak digital science Image Station 440CF. Protein loading and signal intensity was controlled by normalizing each sample to the aforementioned positive control, PE01-WWOX as previously described [10,12].

### Ovarian tissue microarrays

The MD Anderson Cancer Center ovarian TMA was prepared by D.R. at the J.L laboratory [17] with samples from 441 patients with primary epithelial ovarian cancer that had undergone surgery at M. D. Anderson Cancer Center between 1990 and 2001. Follow-up information was updated through June 2003. Histopathologic diagnoses assigned at the time of treatment were based on World Health Organization criteria, each sample was reclassified by grade based on the Gynecologic Oncology Group (GOG) criteria [18] and each case was also classified by disease stage according to the International Federation of Gynecology and Obstetrics (FIGO) system [19]. The appropriate institutional committee approved use of human tissue blocks and clinical records reviews. Each core was scored individually and the results are reported as the mean of at least two replicate core sample measurements [20].

We also used a second ovarian TMA set generated at the Fox Chase Cancer Center (FCCC) tumor bank by R.P. at the A.J.P.K laboratory. This TMA set included a total of 86 ovarian tumors and 21 normal ovarian samples. Cores of normal ovaries were used as positive controls. Only primary mucinous carcinomas of the ovary where included on this cohort of patients. Selection criteria was based on histopathological diagnosis made by a trained pathologist on whole sections, histochemical stainings, immuhohistochemical staining, and clinical assessment of the patient. By pooling the TMA sets from both institutions we were able to successfully analyze in duplicate WWOX protein staining from a total of 444 invasive surface epithelial derived primary ovarian carcinomas that included Serous (n = 375), Endometroid (n = 40), Mucinous (n = 10) and Clear Cell Carcinoma (CCC) (n = 19) histological subtypes. A subset of these cases (n = 323) was successfully processed and analyzed for allowing comparative analysis of WWOX protein expression with ER and PR status.

### Immunostaining method

Anti-WWOX immunostaining was performed as previously reported [12]. Mouse monoclonal antibodies for ER, NCL-ER-6F11 (Novocastra, UK) and PR, NCL-PGR-312 (Novocastra, UK) were used according to manufacturer recommendations. The anti-PR antibody used in these studies is able to recognize only the A isoform of this receptor [21].

### Evaluation of immunohistochemical staining

Staining intensity was measured using a Chromavision Automated Cellular Imaging System (Chromavision^® ^ACIS^®^, San Juan Capistrano, CA) as previously described [12].

The methods for scoring cytoplasmic or nuclear staining are based on different program functions of the ACIS^® ^and are the recommended by the manufacture. For anti-WWOX staining measurements we employed the generic DAB software application provided by ACIS. The area of each individual TMA core was considered for the measurements in its totality. The software determines brown intensity (i.e. positive stained cells) regardless of the area covered by the positive cells. The cutoff to differentiate positive and negative staining was determined to be a mean intensity of 63 (arbitrary staining intensity units, s.i.u.) over a total of 255 (color saturation scale). Therefore, cores with values = 63 s.i.u were 'negative' for WWOX immunostaining, i.e. no brown staining observable. Values between 63 and = 65 s.i.u. were considered to be in the 'weak/low' staining category, i.e. barely visible brown staining, 65 to 81 s.i.u were considered 'moderate' and cores with staining intensity values > 81 s.i.u were considered to fall in the 'strong' staining category. For sake of simplicity cases were classified in two groups: a) negative + low = Negative-Low WWOX group, i.e. any core with a value = 65 in staining intensity and b) moderate + strong = High WWOX group, i.e. any core with values above 65 in staining intensity. The Negative/Low WWOX group was composed of 79% (105/133) completely negative cases and 21% (28/133) weak cases. The mean WWOX intensity staining for normal ovarian surface epithelium (OSE) fell in the moderate category (mean value 70 ± 4).

### Anti-ER and PR antibodies

For performing anti-ER and anti-PR determinations we utilized the nuclear antigen software application provided by ACIS. Only areas of tumor in each TMA core were considered for measurements. The free form drawing tool provided by the software was used to select tumoral areas and to exclude other structures. A final score was determined by the percentage of brown stained nuclei (i.e. positive cells) over the total of tumor cell nuclei measured (i.e. positive plus negative cells). Only cores with at least 10% tumor were included in the analysis. For the receptor analyses, positivity of nuclear staining was defined as follows: weak > 5% stained nuclei, moderate 5–40% stained nuclei and strong >50% stained nuclei.

### Statistical methods

Univariate analysis of clinical-pathological parameters based on WWOX staining was determined by χ^2 ^test with Yates' correction. The basic significance level was fixed at *p *< 0.05 and all data was analyzed using SPSS statistical software (Version 11.0; SPSS Inc., Chicago, IL).

## Results

### Western blot analysis

**Figure 1:**
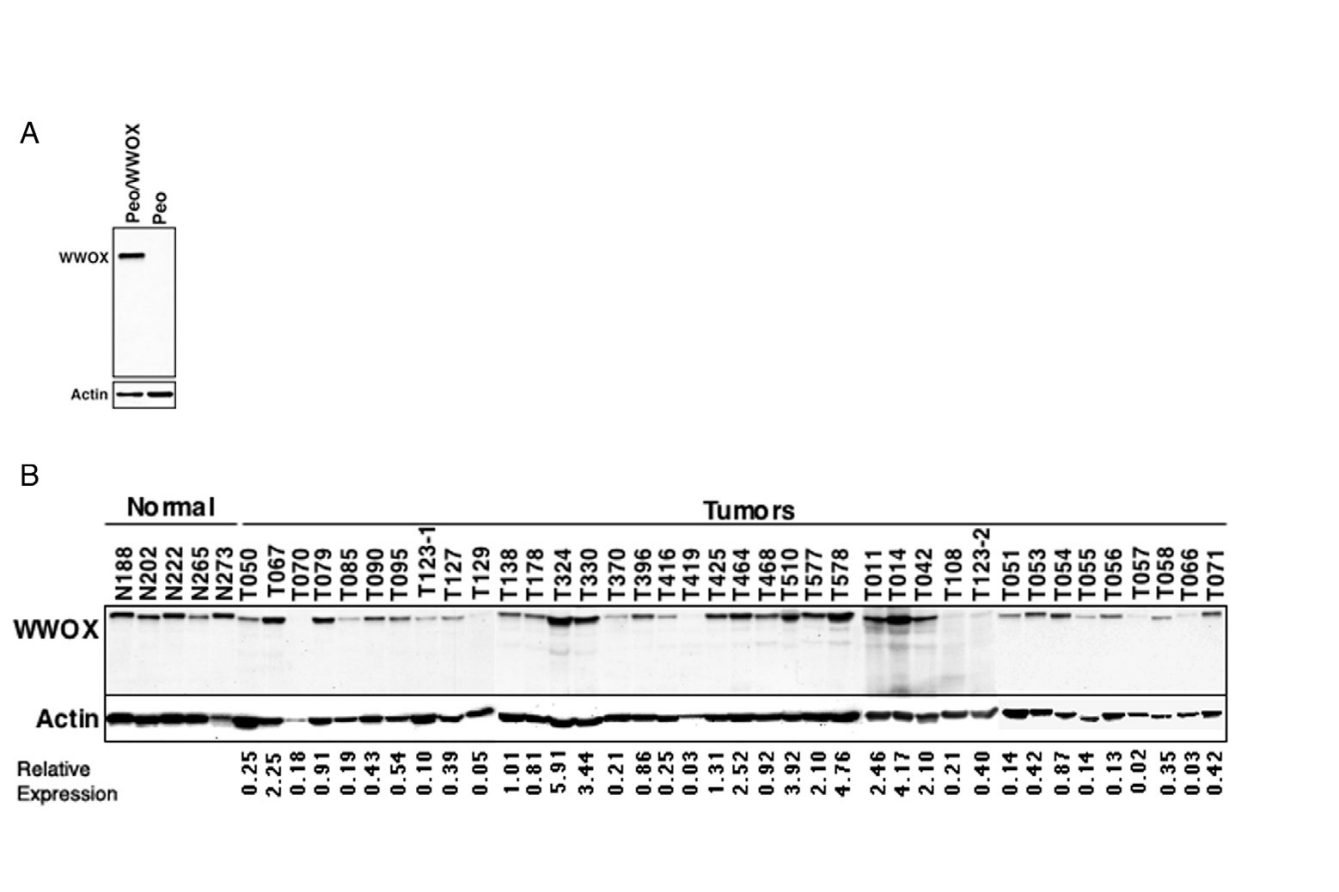
WWOX protein expression in normal ovary and ovarian adenocarcinomas as determined by immunoblot analysis. A) Total protein extracts from PEO1 cell lines transfected with an empty vector or a WWOX expressing vector were analyzed by immunobloting with the anti-WWOX antibody. Note: no immunoreactive bands are observed in the vector transfected cell line. B) WWOX protein expression was determined by immunoblotting of total protein extracts from 38 ovarian carcinoma samples. Five normal ovarian tissue extracts are shown on the first five lanes. Quantitation of WWOX protein expression. Autoradiographs of WWOX and actin were digitized using the Kodak digital science Image Station 440CF. WWOX expression in each sample was normalizated to actin to correct for loading differences. In turn these numbers obtained from each tumor were normalized and expressed as relative to the normal ovarian values (i.e. Relative Expression).

Immunoblotting analysis demonstrated that full length WWOX protein was the predominant WWOX isoform expressed in both normal ovary and ovarian carcinomas (Figure 1B). The anti-WWOX antibody used was raised against the WW domains [2] and should detect all potential WWOX isoforms. The specificity of this WWOX antibody in the immunoblotting analyses was demonstrated by observing no immunoreactive products in cell extracts from the PEO1 ovarian carcinoma cell line used as negative control (Figure 1A). In addition, as a positive control PEO1 cells were stably transfected with a WWOX expression vector (Figure 1A).

Normal ovarian tissues displayed a consistently strong WWOX specific signal while WWOX protein expression levels were extremely variable among tumor samples. Some tumors displayed barely detectable, if any, WWOX protein when compared to normal tissue, e.g. T108 while other samples had significantly higher WWOX levels e.g. T578 in Figure 1. We concluded that 26% (10/38) of tumors over-expressed WWOX, 34% (13/38) expressed normal WWOX levels and 40% (15/38) of tumors had levels lower than 50% that observed in normal ovary.

### Pattern of WWOX staining in normal ovary

Initially we characterized the cellular localization of WWOX protein expression in normal ovaries by IHC using the antibody described in the previous section. The specificity of the WWOX antibody for IHC analysis was validated by observing that the immunoreactivity was abolished by pre-absortion to the recombinant GST-WWOX fusion protein used to raise the antibody (data not shown).

**Figure 2:**
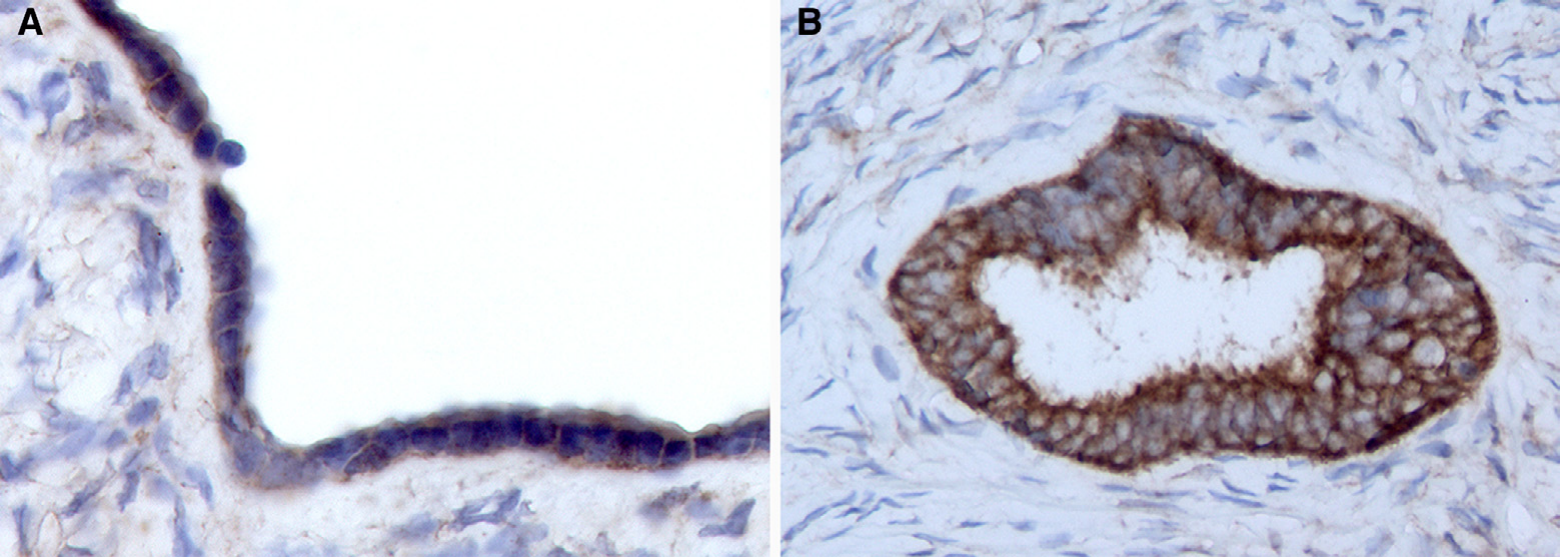
WWOX immunohistochemical staining of normal ovary. A) Representative photomicrograph (20X) of normal ovary displaying positive staining in ovarian surface epithelial cells. B) Photomicrograph (20X) showing strong WWOX inmunostaining localizing to the cytoplasm of inclusion cyst epithelial cells.

The analysis of TMA cores and whole sections of normal human ovary showed that WWOX protein is constitutively expressed at high levels in normal OSE cells (Figure 2A), in epithelial inclusion cysts within the cortex (Figure 2B) and in scattered granulosa-lutein cells surrounding aged corpora lutea or corpora albicans (not shown). Positive staining was also detected in one observed Walthard nest within the ovarian hilus and in a few stromal cell patches likely related with hormone production. In all cases the inmunostaining had a homogeneous and diffuse pattern that was localized to the cytoplasm.

### WWOX staining in ovarian carcinomas

WWOX expression was highly variable among ovarian tumors (n = 444). WWOX staining intensity was determined in all four major surface epithelium derived ovarian carcinoma histotypes, i.e. Serous, Endometroid, Mucinous and Clear Cell (Table 1).

**Figure 3:**
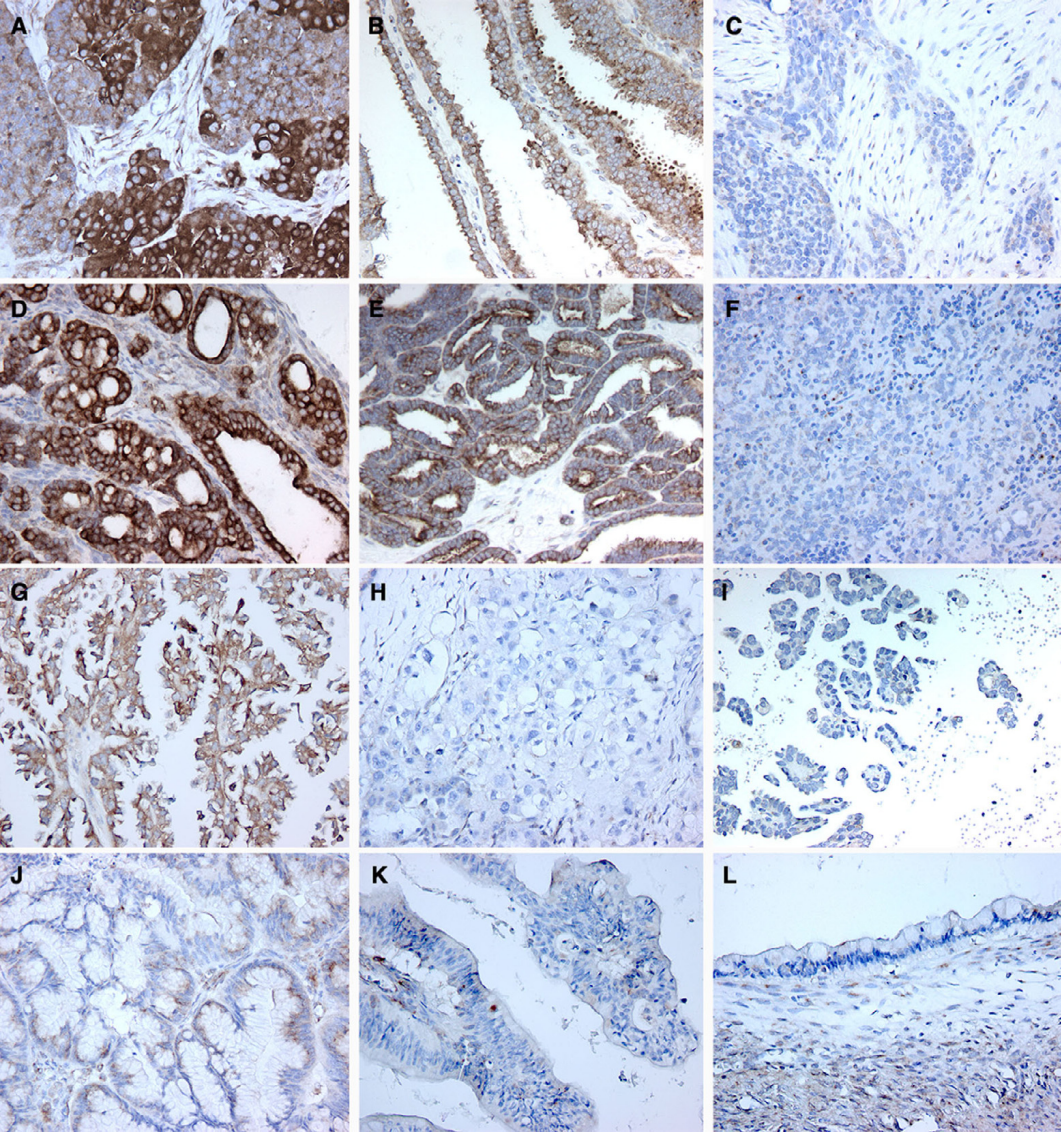
WWOX inmunohistochemical staining in ovarian carcinoma samples by histotypes. A-C. *Serous ovarian carcinomas*. A, Strong-Moderate. Note heterogeneity in staining intensity pattern in this tumor sample; B, Moderate. Note predominance of apical staining in this papillary serous ovarian carcinoma; C Weak-Negative, WWOX lack of staining observed in approximately 30% of serous carcinoma cases. D-F. *Endometroid ovarian carcinomas*. D and E positive WWOX cytoplasmic staining and F negative staining. G-I. *Clear Cell ovarian Carcinomas*. G, representative photomicrograph of one of a moderately WWOX positive CCC case, while H and I, represent typical negative cases. J-L. *Mucinous ovarian carcinomas*, J, representative mildly positive case and K representative mucinous carcinoma of the endocervicoid subtype with demonstrating no WWOX staining. L, mucinous carcinoma of the intestinal subtype also negative for WWOX staining.

Thirty percent of ovarian carcinomas (133/444) showed loss of WWOX protein expression while 70% (311/444) demonstrated positive staining including cases with very strong staining (Figure 3A–I) (Table 1). Among serous carcinomas, 29% of the cases (109/375) fell in the negative/low group and 71% (266/375) stained moderately or strongly positive. The most typical WWOX staining pattern observed for all histotypes was cytoplasmic and diffuse, no tissues demonstrated any nuclear staining. Interestingly, some of the carcinomas showed a distinctive strong cell membrane staining pattern (Figure 3). Other tumors displayed a predominantly apical border staining, in conjunction with staining of what appeared to be luminal secretions (Figure 3B). Among endometroid carcinomas 23% of the cases (9/40) were negative/low and 77% (31/40) stained moderately or strongly positive for WWOX expression. This histological subtype showed a diffuse and apical cytoplasmic pattern of protein expression (Figure 3D, E). Among the CCC group, 42% of the cases (8/19) were negative/weak while 58% (11/19) stained moderately or strongly for WWOX (Figure 3G–I).

Within the mucinous carcinoma group, we observed that most tumors (7 out of 10 cases) did not stain for WWOX expression. Furthermore the three positive mucinous carcinomas were clearly below the mean value for normal OSE. The immunoreactivity localized to the cytoplasm as in the other histotypes but in a very specific perinuclear fashion (Figure 3J). The high rate of absence of WWOX protein expression among the mucinous carcinoma group was statistically different compared to the other three histotypes (p = 0.0131, Table 1).

### WWOX staining analysis according to stage and grade

WWOX expression was next correlated with tumor Stages (FIGO). We observed that 23% of Stage I (7/31), 29% of Stage II (8/28), 31% of Stage III (74/242) and 48% of Stage IV (31/65) tumors showed negative/low WWOX protein expression. We detected a statistically significant trend between the decrease in WWOX expression and more advanced FIGO stages (-b = -0.135; *p trend= *0.007, Table 2). No correlation was found between tumor grade (GOG) and WWOX staining (p = 0.707, Table 2).

### WWOX staining analysis according to relapse and survival

**Figure 4:**
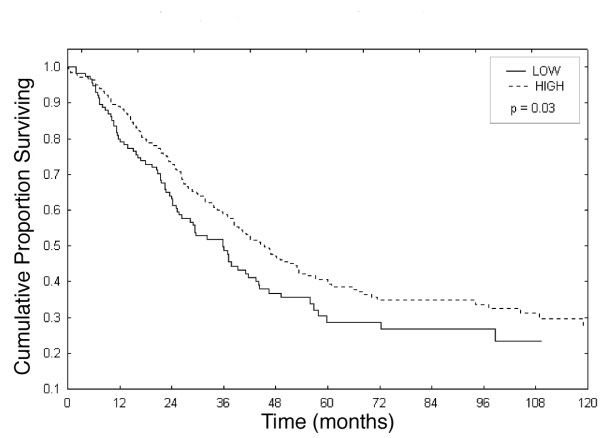
Kaplan Meier Plot analysis showing the pattern of Overall Survival relative to WWOX protein expression.

An analysis of WWOX protein expression relative to disease relapse was performed in a subset of cases from the MDACC TMA (n = 354). Between relapsing and non-relapsing cases no statistical difference was observed regarding WWOX expression (p = 0.094) (Table 2). Among cases with progressive disease, 46% (27/59) showed negative/low WWOX levels of expression while 54% (32/59) fell in the positive category. It was possible to evaluate survival in cases from the MDACC group as shown by the Kaplan-Meier plot (Figure 4). Importantly, we observed a statistically significant correlation of WWOX expressing cases with longer overall survival and loss of WWOX protein expression correlated with shorter overall survival (p = 0.03).

### WWOX staining analysis in correlation with ER and PR status

We analyzed the association of WWOX with ERα and PR status in ovarian carcinoma cases (n = 323). When ER status was correlated with WWOX expression levels no significant differences were found (Table 2). On the other hand, a statistically significant correlation was observed between WWOX expression and PR expression (p = 0.008, Table 2). In the WWOX negative cases 85% (70/82) were PR negative while only 15% (12/83) were PR positive.

## Discussion

Since our original cloning of *WWOX *[1] abundant evidence has accumulated indicating that this gene likely plays a role in either tumor initiation or progression in various neoplasias including ovarian cancer [3-12]. WWOX spans the second most common chromosomal fragile site, FRA16D. This genomic region is prone to chromosome breakage, recombination and gross rearrangements. Multiple ovarian carcinoma studies have demonstrated high frequency of allelic losses for specific chromosomal regions on 16q [22] and since the losses have been found both in low and high-grade tumors it has been proposed to be an early event in ovarian carcinoma development [23]. Loss of heterozygosity at 16q23.2-q24.2 has been correlated with ovarian carcinoma metastasis and advanced tumor stages [24]. Comparative Genomic Hybridization (CGH) studies confirmed those observations also indicating high frequency of 16q21-q24 genomic losses in advanced invasive ovarian carcinoma stages [25,26]. Taken together the data indicates that a gene, likely *WWOX*, located at 16q23-24 is associated with a worse prognosis in ovarian cancer.

In this study WWOX protein expression in normal ovary was predominantly observed in the OSE and epithelial inclusions, both sites proposed to be where ovarian epithelial neoplastic transformation originates. Because of this finding together with the observations of Paige (2001) [4], that ovarian carcinoma cell lines have high frequency of genomic losses affecting the *WWOX *gene including homozygous deletions, we considered it of relevance to analyze WWOX protein expression among the four major surface epithelial derived ovarian carcinoma histotypes. Our analysis by immunoblotting and IHC demonstrated that about one third of ovarian carcinomas displayed extremely reduced or absent WWOX protein expression.

It is worth noting that in all instances, normal and tumor, WWOX staining was exclusively cytoplasmic and in some serous carcinomas staining was also localized to the apical cell membrane or luminal border. This is similar to our observations in breast tumors [12], where WWOX was always cytoplasmic without nuclear staining. This is also in agreement with observations from other groups [11]. This is in stark contrast with Watanabe (2003) [27] who observed that some cells of normal and tumor breast and gastric cases had WWOX protein expression localized to the nuclei. Possibly this discrepancy is due to the use of different antibodies.

In this ovarian cancer study, we also observed that Serous and Endometroid histotypes express normal or high WWOX but two rare but well described ovarian carcinoma histotypes, mucinous and clear cell (28,29,30), demonstrated a higher frequency of loss of WWOX protein expression. This association was more significant for the mucinous type, for which we observed that 70% (7/10) of cases were devoid of WWOX protein expression. Eventhough a small number of mucinous cases were studied the overall high frequency of decreased or loss of WWOX in these tumors as a group is intriguing. Borderline significance was found for the pure CCC for which 42% of cases demonstrated no or very low WWOX protein expression (Figure 3H versus 3G).

Primary mucinous ovarian tumors have been shown to carry a higher frequency of K-ras mutations as one of the few genetic hallmarks able to distinguish it from other histotypes, while CCC are characterized by a lack of p53 mutations. Mucinous carcinomas and CCC, has been associated with a higher frequency of resistance to chemotherapy (31). Interestingly, in our study, we observed a statistically significant trend between loss of WWOX expression and worse patient outcome. Patients whose tumors expressed WWOX at moderate or high levels fared better than those with low expression of this enzyme (Figure 4). We also found a statistically significant correlation with loss of WWOX and stage IV at initial diagnosis (FIGO). The association with overall survival could be due to an increased frequency of loss in higher stage tumors that are associated with a poorer outcome or alternatively due, in part, to an increased frequency of clear cell and mucinous tumors which have a lower likehood of responding to therapy [31]. It was also of interest that we observed a positive correlation between WWOX and PR expression. In contrast to our previous observations in breast cancer in which we observed a strong correlation between ER and PR with WWOX expression [12], in this ovarian study, only PR was associated with WWOX expression (Table 2). Specifically PR negative ovarian carcinomas also lacked WWOX expression. Progestins acting through the PR have been postulated to be protective for ovarian carcinoma development [32,33] due to a postulated ability of inhibiting cell proliferation and inducing apoptosis (34,35). We noticed that all (100%) of our analyzed mucinous carcinomas were PR negative which agrees with the literature regarding absence of PR expression in mucinous tumors [36,37] while 90% of CCC were PR negative. These correlations raise the question of whether the observed positive association between PR loss and WWOX loss is a consequence of the predominance of WWOX loss in the two aforementioned histotypes, rather than a direct mechanistic association between WWOX and PR. Changes in progesterone signaling and regulation of progesterone-responsive genes can be of critical significance in the ovarian tumorigenic process by itself [38]. The opposite could be true as well i.e. that high levels of WWOX expression is associated with PR expression and as a consequence related to the putative protective effect of progestins that in turn could be associated with more favorable patient outcomes.

## Conclusion

We provide evidence that WWOX protein expression is frequently altered and highly variable in ovarian carcinomas. The loss of WWOX expression shows association with Mucinous and CCC histotypes more than the others and also shows a tendency with PR negative expression. Significantly, reduced WWOX protein expression correlates with less favorable outcome.

## Competing interests

The author(s) declare that they have no competing interests.

## Authors' contributions

CMA conceived, coordinated, designed the study, interpreted the data and revised the manuscript. MIN coordinated, performed pathological and immunohistochemical study, interpreted the data, drafted and write the report.DR constructed M.D. Anderson Cancer Center Tissue Microarray and interpreted the data. JHLM participated in preparation of the antibody, Western Blot analyses, interpretation of the data and critically reviewed the manuscript. MCA performed statistical analysis, figures and tables and interpreted the data. HK carried out Western Blot analyses. RP constructed Fox Chase Cancer Center Tissue Microarray. AJPKS, AG, JL and GM provided tumor samples, interpretation of the data and critically reviewed the manuscript. All of the authors have read and approved the final manuscript.

## Pre-publication history

The pre-publication history for this paper can be accessed here:



## Figures and Tables

**Table 1:** WWOX intensity staining according to the four major epithelial derived ovarian carcinoma histotypes.

***Histology***	***WWOX intensity***		***Total***
		
	***Low (Negative/Weak)***	***High (Moderate/Strong)***	
**Serous carcinoma**	109/375 (29%)	266/375 (71%)	**375**
**Endometroid carcinoma**	9/40 (23%)	31/40 (77%)	**40**
**Clear cell carcinoma**	8/19 (42%)	11/19 (58%)	**19**
Mucinous carcinoma *	7/10 (70%)	3/10 (30%)	**10**

**Total**	**133/444 (30%)**	**311/444 (70%)**	**444**

* (χ^2 ^= *8.67; p = 0.0131)*

**Table 2:** Clinical parameters and steroid receptor status analyzed in correlation with WWOX staining.

***Clinical parameters***	***WWOX intensity***		***Total***	***p value***
			
	***Low (Negative/Weak)***	***High (Moderate/Strong)***		
**Grade (GOG)**				
1	4 (23%)	13 (77%)	**17**	*p = 0.707*
2	9 (33%)	18 (67%)	**27**	
3	107 (33%)	215 (67%)	**322**	

**Stage (FIGO)**				
I	7 (23%)	24 (77%)	**31**	*p = 0.007*
II	8 (29%)	20 (71%)	**28**	
III	74 (31%)	168 (69%)	**242**	
IV	31(48%)	34 (52%)	**65**	

**Relapse**				
No	31(32%)	66 (68%)	**97**	*p = 0.09*
Yes	61(31%)	137(69%)	**198**	
Progressive disease	27(46%)	32 (54%)	**59**	

**ER**				
Negative	22 (25%)	66 (75%)	**88**	*p = 0.984*
Positive	59 (25%)	176 (75%)	**235**	
**PR**				
Negative	70 (29%)	170 (71%)	**240**	*p = 0.00*
Positive	12 (14%)	71 (86%)	**83**	
